# An LLM-based methodology for the automatic detection of bias in the DuoWikiBias corpus

**DOI:** 10.3389/frai.2026.1791624

**Published:** 2026-05-13

**Authors:** Karla Salas-Jimenez, Sergio-Luis Ojeda-Trueba, Gemma Bel-Enguix, Edgar Lee-Romero, Francisco F. López-Ponce

**Affiliations:** 1 Universidad Nacional Autónoma de México, Ciudad de México (CDMX), Mexico City, Mexico; 2 Posgrado en Ciencia e Ingeniería de la Computación, Universidad Nacional Autónoma de México (UNAM), Ciudad de México (CDMX), Mexico City, Mexico

**Keywords:** bias detection, ensembles, generalization of models, gradient ascent, LLM, prompt engineering, unlearning, Wikipedia

## Abstract

Bias detection remains a challenge in Natural Language Processing, particularly in non-English contexts, due to the conceptual ambiguity of bias and the scarcity of annotated resources. This study addresses the lack of Spanish-language resources by investigating the automatic detection of framing, epistemological, and demographic^1^ biases. We introduce DuoWikiBias, a novel parallel corpus derived from Wikipedia for Spanish bias classification. We evaluate Large Language Models (Llama and Gemma) using advanced prompting techniques—CARP and Metacognition—combined with a Gradient Ascent unlearning method to refine model attention. Their performance is compared against classical approaches, including logistic regression with S-BERT embeddings and linguistic features. Results show that advanced prompting substantially improves performance over simple instructions, while the best overall performance (F1 = 0.796) is achieved by combining CARP-based features with Gradient Ascent and a Support Vector Machine classifier. These findings suggest that LLMs are effective for bias-aware representation learning, but hybrid approaches with traditional classifiers remain competitive. This work provides both a validated dataset and a methodological framework for bias detection in Spanish NLP.^2^

## 1 Introduction

Bias detection remains a challenging task in Natural Language Processing (NLP), primarily due to the concept's inherent fuzziness: (1) Pryzant et al. (2020) conceptualize bias as a form of inappropriate subjectivity, which is defined as language that introduces attitudes via framing, presupposing truth, or casting doubt in a case when it should be free from opinion.^3^ In a similar vein, Recasens et al. (2013) contrasts biased language with text that has a neutral or objective point of view.^4^ Blodgett et al. (2020) criticizes this vague definition, saying that *the term bias (..) is used to describe a wide range of system behaviors*, *even though they may be harmful in different ways, to different groups, or for different reasons*. (2) The introduction of bias can be conveyed through very subtle strategies. For example, it can be manifested at the lexical level, where meaning is conditioned by contextual and connotative distinctions:

(1) *He killed/murdered three police officers*.

It may also arise through the use of adverbs:

(2) *The reproductions were fantastically/accurately crafted*.

Or through syntactic framing, where the structure of the sentence influences interpretation:

(3) *The protester was shot by the police* vs. *The police shot the protester*.

The identification of bias is of critical importance in two principal domains: the development of neutral computational technologies through the use of unbiased corpora, and the creation of bias detection tools to assist professionals in fields such as scientific research and legal documents.

From an NLP perspective, the problem has been addressed using supervised machine learning techniques to detect different types of bias. Most contributions in this area are in English, with a remarkable lack of experimentation in other languages, including Spanish. In order to mitigate biases and create models that are as neutral as possible, we highlight two approaches that have been proposed in recent years: (a) Training models with unbiased data. (b) Unlearning techniques.

Training models with unbiased data would be the ideal solution for bias in transformer-based models. However, given the rapid pace of model development and the vast amount of training data involved, manual expert inspection for bias is infeasible; consequently, this process must be automated.

The present work addresses the bias detection problem with LLMs from different perspectives. First, we created the DuoWikiBias corpus, a revised and validated Spanish translation of the WIKIBIAS corpus, designed to detect and classify biases. Second, we propose a strategy for bias detection based on the study of prompts that assist in reasoning for this task. Finally, we apply an unlearning technique, gradient ascent, to the biased sentences of DuoWikiBias and test the same prompts with the hypothesis that performance will improve after the application of the unlearning algorithm.

This paper is structured as follows. Section 2 introduces the types of biases covered in this paper. It then discusses the state of the art in bias detection and unlearning algorithms, with a particular focus on existing corpora and methodological approaches. Section 3 details the construction and curation of the DuoWikiBias corpus, while Section 4 describes the methodology. Section 5 presents and analyzes the results. Finally, the paper closes with the conclusions and future work in section 6, and the limitations of this work in section 7.

## 2 Related work

This section reviews the state of the art in resources and methods relevant to our study. First, we survey studies that have developed corpora or enhanced existing ones for bias identification. Subsequently, we examine prompting strategies for Large Language Models (LLMs) designed to improve reasoning and task performance. Finally, we discuss unlearning algorithms that have demonstrated efficacy and are pertinent to our research.

### 2.1 Types of bias

The term bias encompasses a wide range of types. For example, there are cognitive biases, which affect our behavior and decision-making processes, and cultural biases, which reflect our inclinations toward shared personal beliefs (Baeza-Yates, 2018). There are also more specific kinds of bias; for instance, Hovy and Prabhumoye (2021) identify five types of bias within NLP:

1. Data: Many corpora are created from online sources written by relatively homogeneous groups of people (in terms of age, education, social class, and race) at specific points in time.
2. Annotation: It can introduce multiple types of bias. Annotators often share similar demographic traits, and errors can occur due to distraction, disinterest, or disagreement when multiple labels are plausible. Recent work addresses this through soft labels, which take annotator disagreement into account and assign multiple trained annotators to each instance.
3. Representation: Word embeddings inherit and reflect social biases, such as gender stereotypes (Bolukbasi et al., 2016).
4. Models: Studies such as Garrido-Muñoz et al. (2023) and Sant et al. (2024) show that models like BERT (Vaswani et al., 2017; Devlin et al., 2019), RoBERTa (Liu et al., 2019), and LLMs contain social and linguistic biases. Some of these biases may even be amplified during training due to loss functions that exploit spurious correlations, causing models to provide correct answers for the wrong reasons. Another issue is that models are often forced to make predictions, even when lacking sufficient information.
5. Research design: English dominance and topic preference create inequality in scientific progress.

This work uses the categorization proposed by Pryzant et al. (2020), which is an extension of the typology introduced by Recasens et al. (2013).

- Framing bias. It refers to the deliberate selection and structuring of elements (in this case, linguistics elements) from reality to construct a narrative that emphasizes certain meanings or relationships, guiding the audience toward a specific interpretation (Entman, 2007). This is shown through phrases or words linked to a particular point of view using intensifiers and one-side terms that usually reveal the stance of the author (Recasens et al., 2013).

- Subjective intensifiers are lexical elements (typically adjectives or adverbs) that amplify the expressive or evaluative force of a phrase or proposition, introducing an element of subjectivity into its interpretation (Recasens et al., 2013).
- One-side terms present only one side of a contentious issue, typically concerning topics like politics where a single event may be interpreted from multiple, often opposing, perspectives (Recasens et al., 2013).

(4) *However, the Swedes were beaten by the dominant Canadian squad in the gold medal match and settled for the silver medal overall*.

The use of the word “dominant” is a subjective intensifiers that enlighten the opinion of the author.

(5) *Other members are the former euro-congressman and vice-president of the European parliament Alejo Vidal-Quadras Roca and Jos Antonio Ortega Lara (well known for having been kidnapped by the terrorist group ETA for more than a year)*.

Here the term “terrorist” could be interpreted as a stance of the author against this group.

- Epistemological bias. This type of bias involves propositions that are widely assumed to be either true or false, which are subtly conveyed in the text through linguistic features that subtly (often through presupposition) focus on the credibility of a proposition. It operates bidirectionally, emerging either when doubt is cast on a proposition generally accepted as true or when an assumption is made about one typically regarded as false (Recasens et al., 2013). Particularly, epistemological bias can be manifested with presupposition, entailment, assertion, or hedging:

This type of bias often introduced through factive verbs, entailments, assertive verbs, and hedges:

1. Factive verbs Presuppose the truth of their complement clause (Kiparsky et al., 1970).
2. Entailment is a directional relationship in which the truth of one expression necessarily follows from the truth of another. The most famous examples are the words *murdered* and *killed*.
3. Assertive verbs. Hooper (1975) defines assertive verbs as those that assert a proposition in their complement clause, where the truth is not assumed, but the certainty depends on the verb. For example, verbs like *claim* or *pointed out*.
4. Hedges serves to weaken one's commitment to a claim, preventing overly confident predictions or assertions (Recasens et al., 2013).

(6) *In 1983 , Noel Tichy recognized that because we are all beings of habit we tend to repeat what we are comfortable with*.

In this case the use of the factive verb “recognized” assumes the truth of the complement *because we are all beings of habit we tend to repeat what we are comfortable with* as a fact.

(7) *To interview Ayatollah Khomeini, she was allegedly forced to wear the chador*.

The adverb “allegedly” suggests that the writer does not believe it happened, the use of this word here function as a hedges.

- Demographic bias. Text with presuppositions about particular population categories or characteristics (Pryzant et al., 2020). Also, about model performance, Gupta et al. (2024, p. 2) mention “A model is biased if it does not perform consistently across all demographic groups.”; also pointing out that this type of bias has the biggest potential to harm real-world scenarios. Based on the definition provided by Gupta et al. (2024), the specific demographic bias includes gender bias (against a particular gender), racial bias (against certain races), ethnic bias (against certain ethnicity) and sexual orientation bias (against certain sexual preferences); but the possibilities of demographic bias is openly wide and can include cultural bias (against particular cultural identities like the urban tribes) or income-based hierarchy (against a particular income like poor populations).

(8) *How should men interact in society?*

This sentence assumes that all people are men, falling in a gender bias.

(9) *Albi Cathedral, formally the Cathedral Basilica of Saint Cecilia (French : Basilique Cathdrale Sainte-Ccile d'Albi), is the most important religious building in Albi, France, and the seat of the Archbishop of Albi (in full, Albi-Castres-Lavaur)*.

The cultural/religion bias is present in this sentence since in Albi could be another important religious centers that are not catholic. The unbiased way could be “[...] the most important Catholic building [...]”

### 2.2 Corpora in English

One of the first corpora for bias identification is the NPOV parallel corpus created by Recasens et al. (2013). The authors consider two types of bias: framing and epistemological bias. This resource, in English, is built from Wikipedia sentences in the wake of reviews that violated the Neutral Point of View (NPOV) policy,^5^ which seeks to prevent articles from containing controversial opinions or statements written as facts and from using non-factual language.

Pryzant et al. (2020) added a third type of bias, demographic bias, and changed the name to Wiki Neutrality Corpus (WNC). More recently, Zhong (2021) identified that the WNC corpus has a series of issues: first, there is a lot of noise in the corpus and some sentence pairs are not related to bias mitigation. They are only style or grammar corrections, even though they are marked as biased.

A second problem emerges within the neutralization mechanism. It is often necessary to make more than one correction in a sentence to remove the bias that was not initially contemplated. Therefore, the authors propose a new corpus to solve these problems: the WIKIBIAS corpus. This resource has a detailed labeling system that indicates the type of bias in each example: framing, epistemological, or demographic.

More recently, Spinde et al. (2021) proposed the BABE corpus, which consists of 3,700 sentences: 1,700 from MBIC (Spinde et al., 2021) and an additional 2,000 on news with controversial topics extracted from 14 US news platforms from January 2017 to June 2020. For each sentence, the BABE corpus indicates the political posture, whether the sentence is biased, and which words introduce this bias.

In addition to other corpora that cover specific types of bias, for example the corpus created by Waseem and Hovy (2016) includes racist and sexist tweets collected from Twitter.

### 2.3 Corpora in Spanish

Several studies have addressed specific forms of bias, with a predominant focus on demographic bias. Notable among these are shared tasks such as EXIST (sEXism Identification in Social neTworks) (Plaza et al., 2024), conducted as part of CLEF, and DETESTS (DETEction and classification of racial STereotypes) (Ariza-Casabona et al., 2022), organized within IberLEF. Both corpora are composed of tweets; however, the former includes one label per annotator to introduce the paradigm of learning with disagreements.

Similarly, Huang et al. (2020) constructed a multilingual Twitter corpus to evaluate demographic bias across several languages, including English, Italian, Polish, Portuguese, and Spanish. Extending this line of research, Neplenbroek et al. (2024) introduced MBBQ (Multilingual Bias Benchmark for Question-Answering), a carefully curated adaptation of the English BBQ dataset extended to Dutch, Spanish, and Turkish, designed to measure social stereotypes shared across these linguistic contexts. In addition, Ratovicius et al. (2024) developed a Spanish-language corpus for detecting gender bias in legal texts.

Beyond social media, news articles have also served as an important domain for bias detection. Monroy-Trujillo and Padilla-Castillo (2025) examined potential biased representations of illegal immigration in Spain across the four most-read online newspapers during the first year of the war in Ukraine. In a similar exploration of online discourse, Kharitonova et al. (2025) created EsCorpiusBias, a corpus derived from the Mediavida forum that captures bias across a wide range of topics, including gaming, technology, and social issues. Finally, the SESGO dataset (Robles et al., 2025) was developed wiht a dataset of biased prompts and responses, focusing on stereotypical generative outputs within the Latin American context.

### 2.4 LLMs and unlearning

Regarding the use of LLMs in several tasks of NLP, some authors have designed specific prompts to improve the reasoning and responses of the models. Sant et al. (2024) used the ‘Let's think step by step' approach (Kojima et al., 2022), which improves LLMs' performance by developing their reasoning in every step of the problem to provide an answer, which is usually called step by step. Chain-of-Thought (Wei et al., 2022) is a few-shot prompting strategy designed to mitigate gender bias in the translation task.

This advancement in reasoning capabilities facilitates tasks such as identifying biases that necessitate contextual analysis. It is important to note that these LLMs, like human experts in bias detection, are not free from bias, as they are trained on data that reflects human biases. In other words, LLMs are capable of providing an answer, but it is important to consider this limitation. This is why the discipline of unlearning has emerged in recent years.

Unlearning seeks to modify the internal weights of an artificial intelligence model with the aim of making the model forget specific values corresponding to sensitive information without losing general functionality (Chen and Yang, 2023; Liu et al., 2025). There are several methods to remove the influence of a specified subset of training data points from trained models. An example is the work of Yao et al. (2024), which shows that using algorithms to forget certain information is more efficient than retraining these models.

Several unlearning algorithms have been applied to different tasks. Examples to address bias mitigation with this approach are Yu et al. (2023) and Dige et al. (2024). Both articles explore the use of Partitioned Contrastive Gradient Unlearning (PCGU), while the second work applies the method to decoder models and Negation via Task Vector.

In addition, the work of Zmigrod et al. (2019) presents a new approach to counterfactual data augmentation^6^ for mitigating gender stereotypes in morphologically rich languages. An unsupervised approach employs dependency trees, lemmas, part-of-speech (POS) tags, and corpus morphosyntactic tags from Universal Dependencies. In Spanish, it reduces gender stereotypes in neural models of language by a factor of 2.5 without compromising their grammaticality.

## 3 Creation of DuoWikiBias corpus

In this section, there will be a discussion about how the WIKIBIAS corpus is composed and the process of creating the DuoWikiBias corpus.

WIKIBIAS^7^ (Zhong, 2021) is a manually annotated parallel corpus of over 4,000 biased and neutralized sentence pairs from Wikipedia edits. This corpus contains annotations of both sentence-level bias types and token-level biased segments, comprising 1,525 single-word and 2,068 multi-word annotations.

Since creating a corpus from scratch is a resource-intensive and time-consuming task, we translated the WIKIBIAS corpus to create a starting point for bias detection and mitigation studies in Spanish.

Then, the translation was made automatically using the DeepL translation tool^8^. Subsequently, we initiated a curation process of each translated sentence, developing a rigorous verification and validation. We carried out in three distinct stages.

1. Revision and analysis of the sentences translated by DeepL to identify concrete mistakes and create a guide^9^.
2. Verification of the quality and alignment of the sentences by two experts following the guide.
3. Final validation of each sentence by a different expert.

This process involved a meticulous examination to ensure the presence of bias in the sentence and whether its neutral pair effectively mitigates any bias. During the curation process for DuoWikiBias, we organized the information from WIKIBIAS as shown in Table 1. We conserve all the previous labels from WIKIBIAS (id, bias_word and type), only making modification in the translation in Spanish. In order to curate the translation of WikiBias to create the DuoWikiBias corpus, we conduct two stages: verification and validation.

**Table 1:** DuoWikiBias corpus structure. In the id column, the suffix _0 indicates the possibly biased sentence and suffix _1 the neutral sentence.

id	Sentence	Bias_words	Type
English			
6_0	... How should men interact in society?	[‘men']	0|0|1
6_1	... How should people interact in society?		0|0|0
Spanish			
6_0	... ?Cómo deben interactuar los hombres en la sociedad?	[‘hombres']	0|0|1
6_1	... ?Cómo debe interactuar la gente en la sociedad?		0|0|0

The structure of the type column represents bias categories: framing | epistemological | demographic.

### 3.1 Verification

The verification stage was conducted by two experts in the fields of translation and linguistics. They were provided with a guide in which three main types of mistakes were identified between the pair of sentences in the original corpus and the translation:

1. Both translated sentences were biased: the neutral sentence in Spanish is still biased after the translation.

WikiBias English file:

(10)       [Biased] *The school has an exceptional sports department; its achievements include …*
       [Neutral] *The school has a strong sports department; its achievements include …*

Both Spanish translated sentences were the same:

(11) *El colegio cuenta con un departamento de deportes excepcional: entre sus logros se incluyen …*

To accomplish a more neutral translation, we change “excepcional” to “sólido” to avoid favoritism.

2. Both translated sentences were neutral: the biased sentence in Spanish is still neutral after the translation of WikiBias in English.

WikiBias English file:

(12)       [Biased] *Melbourne is considered to be Australia's second city, after Sydney*.
             [Neutral] *Melbourne is the second-largest city in Australia, after Sydney*.

Both Spanish translated sentences were the same:

(13) *Melbourne es la ciudad más grande de Australia, después de S*í*dney*.

In English, the biased sentence contains “is considered to be”, which is not in the translation. The correct translation would add “es considerada” in Spanish. This item is biased because this utterance functions as a fact. We change it to “is considered” to avoid any kind of author's beliefs.

3. The sentence structure is confusing or is originally damaged: The bias and/or the neutral original sentence have a confusing configuration or mistake that affects the translation.

WikiBias English file:

(14) *The gaymers community provides a “safe place” to create a “safe place” for LGBT gamers apart from the isolation they feel from both the heteronormative gaming community and the gay community*.

Spanish Translation:

(15) *La comunidad gaymers proporciona un “lugar seguro” para crear un “lugar seguro” para los jugadores LGBT [...]*

Since a word or phrase is repeated, we remove one for the translation: “La comunidad gaymers propone un “lugar seguro” para [...]”

When this stage was finished, a total of 8,190 sentences (4,095 pairs of sentences) were reviewed, and 419 of them were corrected for belonging to one of the cases described above. We also verified the words that introduced a bias in 3,144 sentences.

### 3.2 Validation

After the initial review certain sentences continued to be problematic. Consequently, a process of validation was conducted by an expert in the fields of anthropology and translation, resulting in the identification of four additional cases:

1. Errors in the translation: when a translation does not accomplish the intended meaning of the original English text.

WikiBias English file:

(16) *Up to twenty-four Iraqu*í*es were subsequently killed ..*.

Spanish translation:

(17) *Hasta venticuatro Iraqu*í*es murieron posteriormente ..*.

The verb “killed” has been translated as “died/murieron” which has different connotations and meanings. In the original instance, we state that an individual died as a direct result of another person's actions. In the other case, we are simply indicating the outcome.

2. Repeated sentences: some sentences are very similar, with only a few words added. We also noticed that the bias was very repetitive in other cases. For example, it appeared in sentences containing the word “comedienne” and different awards, such as the “Grammy award”. These sentences are not included in the final version of the corpus.
3. Bias in sentences. In certain instances, the neutral sentence may still exhibit biases derived from the English corpus, which may be reflected in the translated text.

Wikibias English file:

(18)       [Biased] *Other notable individuals such as Dr. Terence Kealey, a clinical biochemist, have written outside of their field of expertise on ADHD*.
             [Neutral] *Other notable individuals such as Dr. Terence Kealey, a clinical biochemist, have written for newspapers on ADHD*.

Although they address the bias outside of their field of expertise, it is evident that the designation of notable at the beginning of the sentence serves to enhance the reputation of the doctor in question. The most appropriate method to solve this issue is to eliminate the persistent biases for the translation of the neutral sentence.

4. New information: This case proved to be the most challenging, as the corpus originates from Wikipedia articles, where additional information is sometimes incorporated into entries to mitigate bias. However, such additions may introduce inconsistencies or inefficiencies, particularly when the reader is not sufficiently familiar with the source article. For instance, while the following pair is labeled as neutral, the second sentence includes supplementary information that could potentially compromise its neutrality.

Wikibias English file:

(19)       [Biased] *All were widely read on both sides of the Atlantic*.
             [Neutral] *All were widely read on both sides of the Atlantic, though The Bravo was a critical failure in the United States*.

As there were fewer than 200 sentences in this case, we decided to remove them from the final corpus; however, we kept a file with these sentences (and those from the other cases).

At the end of the validation, out of 8,190 sentences from the verification step, 980 had issues: 195 contained new information, 619 exhibited bias, 100 had errors in translation, and 66 were repeated sentences. Table 2 presents a summary of the verification and validation stages.

**Table 2:** Summary of verification and validation stages showing the number and percentage of sentences corrected or identified with issues, and the total remaining sentences after each stage.

Stage	Type	Number	Percentage	Final
Verification				
	Total	419	5.12%	8,190
Validation				
	Total	980	11.97%	7,894
	New information	195	2.38%	
	Bias	619	7.56%	
	Translation errors	100	1.22%	
	Repeated sentences	66	0.81%	

### 3.3 Analysis

The final corpus contains 3,947 sentence pairs in both English and Spanish. There are a total of 7,894 sentences, of which 3,070 are biased sentences and 4,824 are unbiased sentences. Also, it is important to note that many sentences can contain more than one type of bias. The distribution of distinct biases is illustrated in Figure 2.

**Figure 1:**
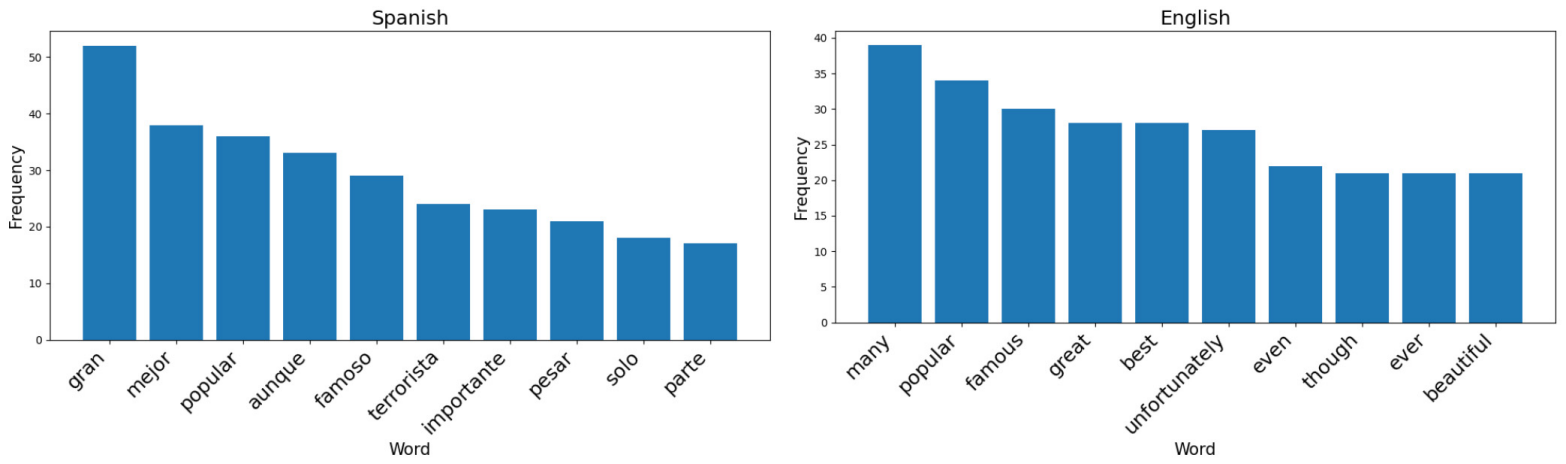
Most common words that introduce bias in DuoWikiBias.

However, it became evident that there are specific words that tend to introduce bias. This phenomenon is illustrated in Figure 1.

**Figure 2:**
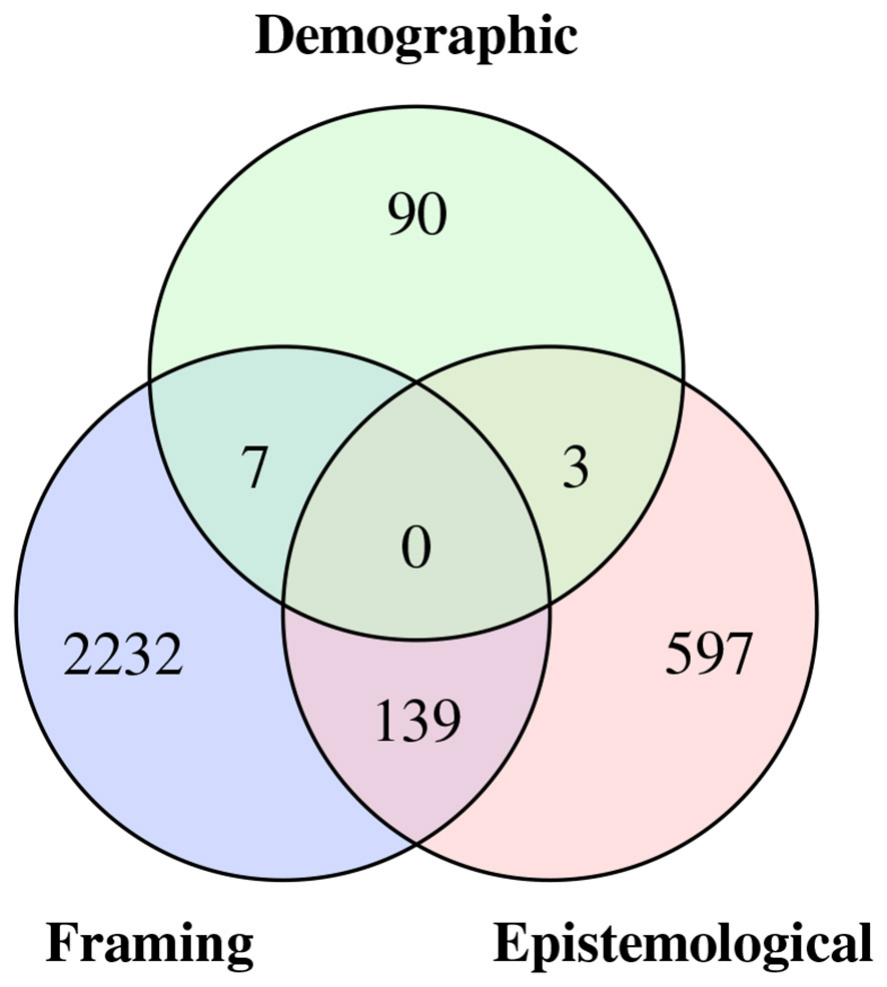
Distribution of types of bias in DuoWikiBias.

Furthermore, note that there are 3 main ways in which the bias can be neutralized:

1. Making a substitution

(20)       [Biased] *The Post-war economic miracle*
             [Neutral] *The Post-war economic growth*

2. Omitting words or phrases

(21)       [Biased] *It was published by the notorious English publisher Edmund Curll*
             [Neutral] *It was published by the English publisher Edmund Curll*

3. Restructuring the sentence

(22)       [Biased] …*Gore's engineering of an ugly “missiles for dead whales” deal with Norway…*
             [Neutral]…*Gore's engineering of what some environmentalists called a “missiles for dead whales” deal with Norway…*

The last case was the most challenging to neutralize.

Another aspect to address is that there are very subtle biases, for example:

(23)       [Biased] *Evolution is the source of the vast diversity of extant and extinct life on Earth*.
             [Neutral] *Evolution may be the source of the vast diversity of extant and extinct life on Earth*.

This kind of sentences are challenging in classification task because the frequency of this types of words that introduces the bias is high and appear in numerous contexts.

## 4 Detection of biases with LLMs

In this section, we use prompts on LLMs to classify texts as biased or not biased. To explain the methodology to achieve this, we divided this section into five subsections: (1) we propose a logistic regression baseline (Section 4.1), (2) we describe the prompts tested and used for classification in LLMs (Section 4.2), (3) we present the gradient ascent algorithm and examples of the model's response (Section 4.3), and (4) we discuss the ensemble between the proposed baseline and the model outputs before and after applying gradient ascent (Section 4.4).

### 4.1 Baseline

To evaluate the effectiveness of LLMs in bias classification, we propose a baseline based on classical Machine Learning algorithms. We trained a logistic regression model using the following concatenated features: (a) Sentence-BERT (Reimers and Gurevych, 2019) specifically the sentence-transformers/paraphrase-multilingual-mpnet-base-v2, each sentence in the corpus was encoded independently into a embedding vector using the encode method, (b) sentiment score derived from the Python package pysentimiento (Pérez et al., 2021), we applied the sentiment analyzer *(task = “sentiment”, lang = “es”)*, which outputs class probabilities for the labels POS, NEG, and NEU. From this output, we sum the probabilities of the POS and NEG classes, and (c) lexicon and syntax based features, we computed the number of adjectives and adverbs in each sentence using the Spanish model from spaCy. Additionally, we calculated the total number of words per sentence that appear in bias-related lexicons compiled by Recasens et al. (2013).^10^

**Figure 3:**
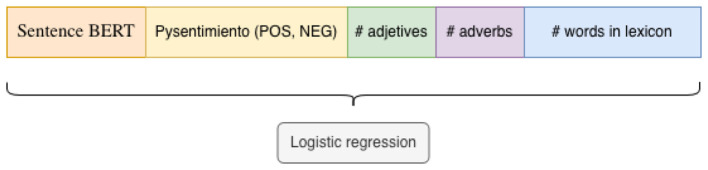
Features employed in the baseline model.

All extracted features (Sentence-BERT embeddings, sentiment score, number of adjectives, adverbs, and lexicon matches) were concatenated into a single feature vector per sentence, as Figure 3 shows. We then trained a logistic regression classifier using the implementation provided by scikit-learn with default hyperparameters.

### 4.2 Prompts

For our initial prompt, we instruct the LLMs to provide an explanation of whether the sentences are biased or not. With this prompt, we consider zero-shot, one-shot, and few-shot approaches, as described by Sahoo et al. (2024). However, we only show the one-shot prompt, as it demonstrated better performance in our preliminary experiments.

**Table:** 

**One-shot** Usuario: Quiero que analices esta oración: < *i*>. A partir de la siguiente oración sesgada, quiero que expliques si la oración anterior también está sesgada. Ejemplo de oración: *El anti-americanismo es un fenómeno reivindicado de discriminación étnica subvertida y abierta hostilidad irracional hacia los Estados Unidos*. Piensa y razona antes de responder. Explica brevemente si la frase está sesgada o no.	**One-shot** User: Analyze this sentence: < *i*>. Please explain whether the previous sentence is also biased, based on the following biased sentence. Example sentence: *Anti-Americanism is a vindicated phenomenon of subverted ethnic discrimination and open irrational hostility toward the United States*. Think and reason before responding. Briefly explain whether the sentence is biased or not.

Subsequently, we considered several prompting strategies, including “Let's think step by step” (Kojima et al., 2022), Chain-of-Thought (Wei et al., 2022), Clue and Reasoning Prompting (CARP) (Sun et al., 2023), and Metacognition (Wang and Zhao, 2024). Based on our experiments, we selected CARP and Metacognition due to their enhanced reasoning capabilities and better performance.

CARP classifies the sentences, considering the definition and types of bias through 4 steps: (1) identifying the clues, (2) reasoning with the given clues, (3) providing a classification that takes into account the previous steps, and (4) identifying the words that introduce the bias, if applicable.

**Table:** 

**CARP:** user: Clasifica la entrada como Sesgada o No sesgada considerando los siguientes aspectos: **Información**: Una oración contiene un sesgo cuando la perspectiva del autor se presenta como verdad absoluta, hay 3 tipos: *Encuadre*- palabras de elogio o desacreditación *Epistemológico*- modifican la credibilidad de una proposición *Demográfico*- relacionado con la raza, el género, etc 1. **Identificación de pistas**: Enumera las palabras clave, frases, información contextual, relaciones semánticas, significado semántico y tonos que apoyan la determinación de algún sesgo en la entrada. 2. **Deducción del razonamiento**: Basado en las pistas identificadas y la entrada, deduce el proceso de razonamiento que apoya la determinación del sesgo de la entrada. 3. **Clasificación de la entrada**: A partir de las pistas y el razonamiento, determina si la entrada es Sesgada o No sesgada. 4. **Identificación de palabras sesgadas**: Si la clasificación es Sesgada, menciona las palabras que introducen el sesgo tomando en cuenta las pistas y el razonamiento. Indique la respuesta final como *Respuesta: (sesgada / no sesgada), Palabras que introducen el sesgo: , Explicación:* **Entrada**: < *i*> assistant: Respuesta:	**CARP:** user: Classify the input as Biased or Unbiased considering the following aspects: **Information**: A sentence contains a bias when the author's perspective is presented as absolute truth, there are 3 types: Framing - words of praise or discrediting Epistemological - modify the credibility of a proposition Demographic - related to race, gender, etc. 1. **Cue identification**: List keywords, phrases, contextual information, semantic relationships, semantic meaning, and tones that support the determination of some bias in the input. 2. **Reasoning deduction**: Based on the identified cues and the input, deduce the reasoning process that supports the determination of the bias in the input. 3. **Classification of the input**: From the clues and reasoning, determine whether the input is Biased or Unbiased. 4. **Identification of biased words**: If the classification is Biased, name the words that introduce the bias taking into account the clues and reasoning. Indicate the final answer as *Answer: (Biased/Unbiased), Words introducing bias:,* *Explanation:* **Input** < *i*> assistant: Answer:

Metacognition (Wang and Zhao, 2024) classifies the input according to a series of steps: (1) to clarify its understanding of the sentence, (2) to make an initial classification and identification of the words that introduce the bias, (3) to evaluate its initial analysis, (4) to confirm its final answer, 5) to tell us its confidence level in the answer, and (6) to give us the final answer. ^11^

For these first experiments we use two checkpoints: google/gemma-2b-it a 2B-parameter architecture from the Gemma 2 series (Gemma Team, 2024), and meta-llama/Meta-Llama-3.1-8B-Instruct, an instruction-tuned 8B-parameter variant from Llama-3.1 (AI@Meta, 2024). We focus on similar LLMs with a low parameter count (< 10B) across different families. Limiting our selection in this manner helps us generate lower bounds of performance for each family of models. In addition, we obtain insight into what strategies work (or not) in the context of bias detection so that further experiments (with bigger models and a bigger use of resources) can be adapted in an optimal way.

For the stage, we used the *generate* method from *AutoModelForCausalLM* with *max_new_tokens=300*, using the standard decoding parameters (e.g., temperature and top-k). A limitation of this setup is that each experiment was run only once. Due to the stochastic nature of the model, results may vary across runs; future work should include multiple runs to assess robustness.

**Table:** 

**Metacognition:** system: Es usted un experto en comprensión del lenguaje natural especializado en detectar sesgos de encuadre, epistemológicos y demográficos. Por favor, asegúrese de que su respuesta es precisa, perspicaz, imparcial y concisa (no más 300 palabras). user: Clasifica la entrada como Sesgada o No sesgada considerando los siguientes aspectos: Para la frase: < *i*>, verifica si contiene algún sesgo. Para realizar esta tarea, siga estos pasos: 1. Aclare su comprensión de la frase. 2. Haga una clasificación preliminar para determinar si la frase contiene algún sesgo. 3. Evalúe críticamente su análisis preliminar. Si no está seguro de su clasificación inicial del sesgo o no, intente reevaluarla. 4. Confirme su respuesta final, mencione las palabras que introducen el sesgo y explique los motivos de su elección. 5. Evalúe su nivel de confianza (0–100%) en su análisis y explique por qué. Indique la respuesta final como *Respuesta: (sesgada / no sesgada), Palabras que introducen el sesgo:,* *Explicación:*. assistant: Respuesta:	**Metacognition:** system: You are a natural language understanding expert specializing in detecting framing, epistemological and demographic biases. Please ensure that your response is accurate, insightful, unbiased and concise (no more than 300 words). user: Classify the entry as Biased or Unbiased by considering the following aspects: For the sentence: < *i*>, check if it contains any bias. To perform this task, follow these steps: 1. Clarify your understanding of the sentence. 2. Make a preliminary classification to determine if the sentence contains any bias. 3. Critically evaluate your preliminary analysis. If you are unsure of your initial bias classification or not, try to reevaluate it. 4. Confirm your final answer, mention the words that introduce bias, and explain the reasons for your choice. 5. Evaluate your level of confidence (0–100%) in your analysis and explain why. Indicate the final answer as *Answer: (biased / unbiased), Words introducing bias: , Explanation:*. assistant: Answer:

### 4.3 Gradient Ascent (GA)

As mentioned at the beginning of this work, previous studies have shown that LLMs contain biases. Our hypothesis for the relatively low performance of these models in classifying whether a sentence is biased, is that they have effectively normalized bias, making it difficult for them to distinguish biased from unbiased content. To address this and make the models more sensitive to bias, we applied gradient ascent, a form of unlearning algorithm.

In order to apply unlearning algorithms, it is necessary to first define the dataset to be forgotten and the dataset to be retained, depending on the specific scenario. In this particular case, the set to be forgotten consists of sentences that contain some form of bias in DuoWikiBias.

The algorithm we chose to try to unlearn bias is gradient ascent over DuoWikiBias sentences containing bias. This is equivalent to the application of the gradient descent algorithm, multiplied by a negative value of one. This is also referred to as the ‘negative gradient' (Yao et al., 2024).

Due to the fact that unlearning algorithms are resource-intensive this experiment was only carried out with the google/gemma-2-2b-it model to make it more accessible to train and to compare before and after prompts. We also quantized the model and used LoRA (Low-Rank Adaptation) (Hu et al., 2022), to make training more efficient with fewer resources. We decided to take the most commonly used parameters to facilitate the comparison of results with the state of the art, which are described in detail in Supplementary material.

It was observed that the responses to the metacognition prompt generally indicated that the sentences did not contain any bias. To mitigate this imbalance, an intermediate step was incorporated.

The response to the prompt metacognition is structured as follows: *Answer: (biased)/(unbiased), Words that introduce bias: (..), Explanation: (...), Confidence level: (...)*. With this in mind, the intermediate step we take is the following: (1) if the answer is *unbiased* but the field *Words that introduce bias:* contains words, we change the original answer from *unbiased* to *biased*, (2) if the answer is *unbiased* and the field *Confidence level* is *80%* or less we change the original answer from *unbiased* to *biased*. As can be seen in Table 3.

**Table 3:** Examples of post-processing rules applied to metacognitive outputs, where *unbiased* predictions are revised based on detected bias words or low confidence (≤ 80%).

Answer	New answer	Bias_words	Conf.	Explanation
Case 1				
*ES: También es conocido por su papel como el sufrido vecino de Victor Meldrew*.				
*EN: He is also known for his role as Victor Meldrew's long-suffering neighbour*.				
no sesgado	sesgado	[sufrido]	95%	El término “sufrido” introduce una connotación negativa que puede inducir una interpretación sesgada.
15-7.4,-52242ptunbiased	biased	[long-suffering]	95%	The term “long-suffering” carries a negative connotation that may lead to a biased interpretation.
Case 2				
*ES: La Universidad de Warwick es un campus universitario de élite en*				
*Gran Bretaña*.				
EN: The University of Warwick is an elite campus university in Great Britain.				
no sesgado	sesgado	[élite]	80%	El término “élite” puede implicar juicios de valor que introducen sesgos epistemológicos o demográficos.
unbiased	biased	[elite]	80%	The term “elite” may imply value judgments that introduce epistemological or demographic bias.

An example of the responses to each prompt is shown below.

Sentence: *?Cómo deben interactuar los hombres en la sociedad?* (How should men interact in society?)

**Table:** 

CARP example: Respuesta: Sesgada Palabras que introducen el sesgo: hombres Explacación: **Información**: La entrada contiene un sesgo encuadrado debido a la perspectiva limitada sobre la interacción de los hombres en la sociedad. La pregunta no considera la perspectiva de las mujeres ni de otras personas que pueden interactuar en la sociedad de manera significativa. **Identificación de pistas**: (1) La palabra clave “hombres” sugiere que la pregunta se centra en la interacción de los hombres en particular. (2) La falta de consideración de la perspectiva de las mujeres y otras personas puede ser vista como un sesgo demográfico. (3) La pregunta no proporciona contexto sobre qué tipo de interacción se refiere (social, laboral, etc.), lo que puede sugerir que se está asumiendo una perspectiva particular. **Deducción del razonamiento**: (1) El proceso de razonamiento que apoya la determinación del sesgo es la asunción de una perspectiva particular (la de los hombres) sin considerar otras perspectivas. (2) La pregunta parece estar basada en una suposición sobre la interacción de los hombres en la sociedad, sin proporcionar evidencia o contexto para respaldar esta suposición. **Clasificación de la entrada**: La entrada es Sesgada debido a la perspectiva limitada,	CARP example: Answer: Biased Words that introduce bias: men Explanation: **Information**: The input contains a framing bias due to the limited perspective on men's interaction in society. The question does not consider the perspective of women or other people who may interact in society in a meaningful way. **Identification of clues**: (1) The keyword “men” suggests that the question focuses on the interaction of men in particular. (2) The lack of consideration of the perspectives of women and other individuals can be seen as a demographic bias. (3) The question does not provide context about what type of interaction it refers to (social, work, etc.), which may suggest that a particular perspective is being assumed. **Deduction of reasoning**: (1) The reasoning process that supports the determination of bias is the assumption of a particular perspective (that of men) without considering other perspectives. (2) The reasoning process that supports the determination of bias is the assumption of a particular perspective (that of men) without considering other perspectives. **Entry classification**: The entry is Biased due to the limited perspective.

**Table:** 

Metacognition example: Respuesta: Sesgada Palabras que introducen el sesgo: los hombres Explicación: 1. Aclaro mi comprensión de la frase: La frase “?Cómo deben interactuar los hombres en la sociedad?” parece preguntar cómo los hombres deberían comportarse o relacionarse con otros en la sociedad. 2. Clasificación preliminar: Mi análisis inicial es que la frase contiene un sesgo demográfico, ya que se enfoca específicamente en los hombres y no menciona a las mujeres ni a otras categorías de personas. 3. Evaluación crítica: Al reevaluar la frase, me doy cuenta de que la pregunta está formulada de manera que asume que los hombres son la única categoría relevante para considerar en este contexto. Esto podría llevar a una visión parcial o sesgada de la sociedad, ya que no se consideran las interacciones y roles de las mujeres o otras personas. 4. Confirmación de la respuesta final: Mi respuesta final es que la frase es sesgada, ya que introduce un sesgo demográfico a través de la palabra “los hombres.” Esta palabra asume que los hombres son la categoría principal a considerar en este contexto, ignorando a las mujeres y otras personas. Nivel de confianza: Mi nivel de confianza en este análisis es del 90%. Estoy seguro de que la frase introduce un sesgo.	Metacognition example: Answer: Biased Words that introduce bias: men Explanation: 1. Clarifying my understanding of the sentence: The sentence “How should men interact in society?” seems to ask how men should behave or relate to others in society. 2. Preliminary classification: My initial analysis is that the sentence contains a demographic bias, as it focuses specifically on men and does not mention women or other categories of people. 3. Critical evaluation: Upon reevaluating the phrase, I realize that the question is phrased in a way that assumes men are the only relevant category to consider in this context. This could lead to a partial or biased view of society, as the interactions and roles of women or other people are not considered. 4. Confirmation of final answer: My final answer is that the phrase is biased, as it introduces a demographic bias through the word “men.” This word assumes that men are the primary category to consider in this context, ignoring women and other people. Level of confidence: My level of confidence in this analysis is 90%. I am certain that the phrase introduces bias.

### 4.4 Ensemble

**Figure 4:**
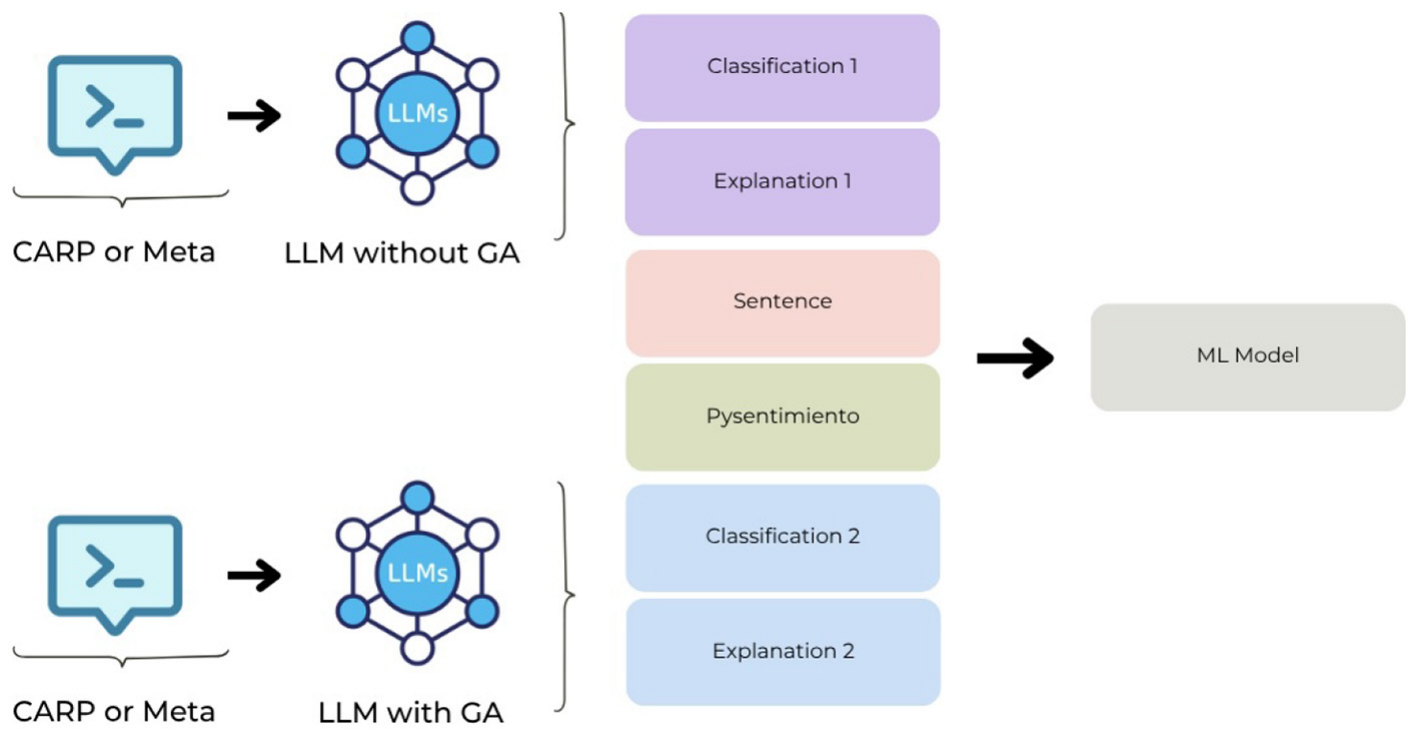
A combination of explanations of different prompts and other features of classic ML models.

In addition, we combined the responses from the two selected prompts for each LLM. We concatenate the embedding of the explanation of each of these responses, the predicted classification of each one, the embedding of the sentence, and the probability that the sentence contains a sentiment using the Python package pysentimiento (Pérez et al., 2021) and pass these features through several classical Machine Learning (ML) models: Logistic Regression (LR), Support Vector Machine (SVC) and Naive Bayes (NB). Figure 4 illustrates this process.

## 5 Analysis and results

The experiments were conducted and evaluated on the Spanish portion of the DuoWikiBias corpus. To assess the generalizability of the best-performing methodology to other corpora, we further evaluated it on two additional datasets:

1. SocialBias, comprising 1,000 sentences (500 biased and 500 unbiased) randomly sampled from the EXIST corpus and 1,000 sentences (500 biased and 500 unbiased) randomly sampled from the DETESTS corpus.
2. WikiEn consisting of 1,000 sentences drawn from the English portion of the DuoWikiBias corpus.

We presented the results in Table 4 and the comparison with other corpora in Table 5.

**Table 4:** Results of the binary classification using different methods. The best result is in bold, and the second-best is underlined.

Method	Accuracy	Precision	Recall	F1 score
Logistic regression				
Baseline	0.610	0.580	0.560	0.550
Llama 3.1				
One-Shot	0.395	0.400	0.400	0.384
CARP (1)	0.521	0.548	0.543	0.515
Meta (2)	0.587	0.575	0.561	0.550
Gemma 2				
One-Shot	0.560	0.280	0.501	0.361
CARP (3)	0.547	0.568	0.554	0.541
Meta (4)	0.513	0.550	0.529	0.466
Gemma 2 + GA				
CARP (5)	0.593	0.591	0.532	0.471
Meta (6)	0.543	0.536	0.536	0.536
Features with combinations				
(1) + (2)+NB	0.634	0.626	0.625	0.626
(1) + (3)+LR	0.560	0.560	0.560	0.560
(1) + (5)+LR	0.781	0.785	0.783	0.781
(2) + (4)+NB	0.603	0.604	0.603	0.602
(2) + (6)+LR	0.766	0.778	0.769	0.764
(3) + (4)+NB	0.698	0.709	0.700	0.695
(5) + (6)+SVC	0.721	0.729	0.719	0.717
(3) + (5)+SVC	**0.797**	**0.811**	**0.800**	**0.796**
(4) + (6)+SVC	0.744	0.749	0.746	0.744

“Meta” refers to the Prompt Metacognition. In the “Combinations” section, numbers refer to prompt results, and letters to classifiers. Complete classifier results are in Supplementary material.

**Table 5:** Results of the best prompts and combinations tested on WikiEn and SocialBias. CARP refers to the selected Prompt.

Method	Accuracy	Precision	Recall	F1 score
CARP Gemma 2				
WikiEn (7)	0.557	0.565	0.565	0.557
SocialBias (8)	0.568	0.615	0.576	0.532
CARP Gemma 2 + GA				
WikiEn (9)	0.590	0.585	0.550	0.517
SocialBias (10)	0.626	0.628	0.620	0.617
Combinations				
(7) + (9)+SVC	0.687	0.671	0.678	0.665
(8) + (10)+SVC	**0.779**	**0.778**	**0.772**	**0.774**

The best result is in bold.

The first relevant point is the importance and power of prompts. If we pay attention to the F1-metric in Table 4 in section *Llama 3.1* and *Gemma 2*, we can notice that CARP and Metacognition have better results than simply instructing the LLMs to provide an explanation of whether the sentence is biased or not, with one example (one-shot).

In addition, it is important to consider the comparison between Llama (8B model size) and Gemma (2B model size). Both models are not as different as we would expect, given the difference in size. This may lead us to believe that it is possible to achieve approximately the same performance in this task with smaller models while using fewer computational resources.

The performance of the CARP and Metacognition prompt exhibited variability. In Llama, metacognition obtained better results, while in Gemma, CARP had the highest performance. Moreover, the Gemma results change slightly when gradient ascent is applied. For the CARP prompt, the F1 score decreases with GA, while it increases for precision and accuracy. In the case of metacognition, performance increases the F1-score.

In an analysis to understand the differences in performance, we noticed that the number of biased and unbiased sentences remains roughly the same. Nevertheless, after applying gradient ascent, several sentences changed their labels, but this did not improve performance in general results.

However, combining the responses from the prompts before and after applying gradient ascent to the model is helpful. In other words, we are applying a kind of ensemble voting while considering the reasons why a certain label was chosen, as discussed in Section 4.4. The best result was achieved with the SVC classifier with CARP on Gemma 2 and CARP on Gemma 2 + GA.

Table 5 shows the generalization of the CARP prompt (the best approach) before and after the GA on SocialBias and WikiEn. The performance remains consistent with SocialBias. In the case of WikiEn, there is a slight decline in performance. This inefficiency was expected due to the training of the model to forget biased sentences in Spanish. Also, the performance decline was not significant since the model was trained in a different language.

Additionally, it is worth noting that these reasoning prompts provided us with more information than we requested, which could be helpful in solving the task. For example, many answers also include the type of bias and the words that introduce the bias.

## 6 Conclusion

This study presents the DuoWikiBias corpus, a resource designed to support bias detection in the development of neutral language systems. In addition, it offers an opportunity to examine how bias manifests across different languages, specifically English and Spanish.

Our results show that solely using prompting strategies on LLMs does not yield optimal performance in this task. However, when LLMs are combined with unbiased methods and traditional machine learning, the efficacy of LLMs is enhanced in this task, and the approach generalizes well to other datasets.

We also find that the construction of a clear, step-by-step prompt that supports reasoning has a greater impact on performance than choosing a more complex model.

Finally, although we achieve an F1 score of 0.796, there is still much to be done in this field. Future work could explore alternative unlearning strategies or algorithms, incorporate granular bias classification, and implement the detection of words that introduce bias using the responses to the proposed prompts.

## 7 Limitations

One of the limitations faced by this work is that DuoWikiBias is a corpus translated from English to Spanish. It has been carefully curated and corrected to accurately capture all biases it original text presents. The final version has been reviewed by three experts. Despite these efforts, it can still contain some non-totally equivalent translations, or the sentences could not cover exactly the same bias than the original English.

In contrast to English, for Spanish there are not as many resources to test the performance of the models on different data, so we cannot be certain of their performance. NLP resources in languages other than English, continue to be scarce, making multilingual tasks complicated.

Moreover, the task presents an additional layer of difficulty. The algorithms and techniques used in the experiments may already be biased. Transformers and LLMs are trained on a large amount of biased data, which means that biases are inherently embedded in these models.

## Data Availability

The datasets presented in this study can be found in online repositories. The names of the repository/repositories and accession number(s) can be found in the article/Supplementary material.

## Footnotes

3
https://www.ischool.berkeley.edu/projects/2022/neutrally-detecting-and-replacing-inappropriate-subjectivity

4
https://en.wikipedia.org/wiki/Wikipedia:Neutral_point_of_view

5 https://en.wikipedia.org/wiki/Wikipedia:Neutral_point_of_view.

6 This involves augmenting a corpus with additional sentences of the class to be contrasted in order to obtain a balanced representation.

7 Code-https://github.com/cs329yangzhong/WIKIBIAS/tree/main. Corpus-https://drive.google.com/drive/folders/1dLKpaVktAojeQ7so1_Seao_e3iLQ-Egy.

8 https://www.deepl.com/en/quality.

9 The guide is available in https://github.com/KarlaDSJ/WikiBiasES.

10 The lexicons were translated into Spanish using the DeepL translator.

11 Since the responses of the Metacognition prompt on Gemma tended to say that most of the sentences were biased we used the degree of confidence in the response; if it was 80% or lower, we changed the label to unbiased.
