# Supplementary material for: An LLM-based methodology for the automatic detection of bias in the DuoWikiBias corpus
Source: Front Artif Intell. 2026 May 13;9:1791624. doi: 10.3389/frai.2026.1791624 (PMC13212246; doi:10.3389/frai.2026.1791624)
Supplement: Supplementary file 2 [file Data_Sheet_2.pdf]

Supplementary Material

1 DATA STATEMENT

We follow the ethical principles outlined in the Belmont Report (National Commission for the Protection of Human Subjects of Biomedical and Behavioral Research, 1979) to ensure that the data collection, translation, and curation processes were conducted with integrity and transparency.

1.1 Curation Rationale

The WikiBiasES corpus was developed to provide a Spanish-language resource for bias detection and mitigation research in NLP. As building a corpus from scratch is resource-intensive, we translated and linguistically adapted the original English WIKIBIAS dataset (Zhong, 2021), a manually annotated collection of over 4,000 pairs of biased and neutralized sentences extracted from Wikipedia edits. This adaptation aims to extend research on linguistic bias to the Spanish language, providing a foundation for cross-linguistic comparisons of bias phenomena and their computational modeling.

1.2 Language Variety

The dataset is written in standard Spanish, aiming a neutral variation in the language. During translation and adaptation, particular attention was paid to maintaining semantic fidelity and bias intensity between the original English sentences and their Spanish counterparts. Lexical variation was preserved where relevant to maintain naturalness and contextual alignment with the original sentences.

1.3 Source and Text Characteristics

The corpus consists of parallel sentence pairs—one biased and one neutralized version—originating from Wikipedia revisions. The Spanish versions were produced through machine translation using the DeepL translation tool[1], followed by manual verification and validation. Each sentence pair retains the original labeling schema, marking the type and degree of bias. Texts are formal and encyclopedic in nature, containing examples of linguistic bias.

1.4 Curators Demographic

The verification and validation processes were conducted by three independent Curators. Two reviewers carried out the initial verification to ensure translation accuracy and semantic alignment between biased and neutralized pairs. A third reviewer, with expertise in anthropology, conducted a secondary validation focused on the cultural and pragmatic nuances of bias expression in Spanish. Also, all were native Spanish speakers with professional backgrounds in translation and linguistics. Table 1.4 summarizes the curators profiles.

1.5 Speech Situation

The original WIKIBIAS corpus was derived from Wikipedia edits, meaning that the text represents encyclopedic, neutral-style writing. The WikiBiasES corpus preserves these characteristics, offering a controlled environment to analyze how linguistic bias is expressed and mitigated in Spanish.

[1] `https://www.deepl.com/en/quality`

Categories	Data
Sex	3 male
Gender	Masculine
Native Language	Spanish
Nationality	Mexican
Residence	Mexico
Education level	University

Table S1. Curators demographic

1.6 Text Characteristics

The final dataset contains 3,947 sentence pairs in both English and Spanish. There are a total of 7,894299 sentences, of which 3,070 are biased sentences and 4,824 are unbiased sentences. After a two-step validation process, 980 sentences were identified as problematic and subsequently excluded or flagged for revision. Specifically, 195 sentences contained additional information not present in the source text, 619 exhibited bias misalignment, 100 presented translation errors, and 66 were repeated. The remaining validated sentences constitute the final version of the WikiBiasES corpus.

All texts were maintained in sentence-aligned format, preserving punctuation, capitalization, and orthographic variation to ensure linguistic authenticity. The dataset covers a wide range of topics, reflecting the diverse content extracted from Wikipedia.

1.7 Recording Quality

All text data were processed into UTF-8 plain text format, ensuring consistency in encoding and tokenization. The corpus was extracted from WikiBias, translated, and validated using standardized digital workflows to guarantee reproducibility. Metadata, curation, and alignment information were stored in tabular parallel format for ease of access and further computational processing.

1.8 Ethical Considerations

All data in the WikiBiasES corpus are derived from publicly available Wikipedia content, ensuring compliance with copyright and data privacy standards. No personal or identifiable information is included. The translation and curation procedures followed ethical research principles outlined in the Belmont Report (National Commission for the Protection of Human Subjects of Biomedical and Behavioral Research, 1979).Curators were fully informed of the study's aims and consented to participate in the verification and validation processes.

2 PARAMETRES

Lora:

```
r = 8
lora_alpha = 16
lora_dropout = 0.05
task_type="CAUSAL_LM"
target_modules = ['q_proj',
                  'o_proj',
                  'k_proj',
```

```
                    'v_proj',
                    'gate_proj',
                    'up_proj',
                    'down_proj']
```

Quantization:

```
load_in_4bit = load_in_4bit,
bnb_4bit_compute_dtype =
           torch.float16,
bnb_4bit_quant_type = "nf4",
low_cpu_mem_usage= True
```

Training GA:

```
training_args = SFTConfig(
      max_seq_length=512,
      report_to='none',
      output_dir="/tmp",
      dataset_text_field="sentence_es",
      per_device_train_batch_size=4,
      per_device_eval_batch_size=4,
      fp16=False,
      bf16=True,
      num_train_epochs=3,
)
```

3 PROMPT PRELIMINARY EXPERIMENTS

In order to select the most effective prompts for this task, four types of prompts are employed: Let's think step by step, Chain-of-Thought (CoT), CARP and Metacognition. The results are present in Table S2.

	**Acc**	**Prec**	**Rec**	**F1**
Let's think step by step	0.50	0.68	0.64	0.49
CoT	0.60	0.60	0.61	0.58
CARP	0.70	0.75	0.78	0.69
Metacogtinion	**0.80**	**0.80**	**0.85**	**0.79**

Table S2. The table displays the preliminary findings from the experiment in which various prompts were used to ascertain the most effective one.

We also show the respective prompts for Let's think Step by Step and CoT:

Let's think step by step

Usuario: Decimos que un texto contiene un sesgo cuando la perspectiva del autor se presenta como verdad absoluta, hay 3 tipos.

Encuadre - palabras de elogio o desacreditación ej. pésima

Epistemológico - modifican la credibilidad de una proposición ej. supuestamente

Demográfico - relacionado con la raza, el género, etc ej. los hombres en lugar de la sociedad

Clasifique el texto en Sesgado o No sesgado. Texto: $< i >$

Let's think step by step

User: *We say that a text contains a bias when the author's perspective is presented as absolute truth, there are 3 types.*

Framing - words of praise or discrediting

Epistemological - modify the credibility of a proposition

Demographic - related to race, gender, etc.

Classify the text as Biased or Unbiased. Text: $< i >$

Let's think step by step.

CoT

User: *Tarea: Clasifique el texto en Sesgado o No sesgado. Texto: En 1994, narró la excelente miniserie de televisión Watergate.*

Assistant: *Respuesta: Sesgada. La palabra excelente nos indica que la serie que sobresale por sus óptimas cualidades, sin embargo no se ofrecen tales cualidades por lo que el termino excelente solo atañe a la opinion del autor.*

User: *Texto: Para entrevistar a Ayatollah Khomeini, supuestamente la obligaron a llevar chador.*

Assistant: *Respuesta: Sesgada. La palabra supuestamente significa que es considerado real o verdadero sin la seguridad de que lo sea, en esta oración el autor esta diciendo que no es cierto que la obligaron restando credibilidad al hecho.*

User: *Texto:¿Cómo deben interactuar los hombres en la sociedad?*

assistant: *Respuesta: Sesgada. Esta oración utiliza Sujeto Universal Masculino al poner hombres dejando de lado a las mujeres, en lugar de la palabra personas que incluye a ambos.*

User: *Texto:* $< i >$

Assistant: *Respuesta:*

CoT

User: *Task: Classify the text as Biased or Unbiased. Text: In 1995 he narrated the excellent TV miniseries Watergate.*

Assistant: *Answer: Biased. The word "excellent" indicates a series that stands out for its excellent qualities, but no such qualities are offered, so the word "excellent" refers only to the author's opinion.*

User: *Text: To interview the Ayatollah Khomeini , she was allegedly forced to wear the chador .*

Assistant: *Answer: Biased. The word "allegedly" means that it is considered real or true without the certainty that it is; in this sentence, the author is saying that it is not true that she was forced to do so, thus diminishing the credibility of the fact.*

User: *Text: ¿It asks such questions as : How should men interact in society ?*

Assistant: *Answer: Biased. This sentence uses the universal masculine subject, putting men and leaving out women, instead of the word people, which includes both.*

User: *Text:* $< i >$

Assistant: *Answer:*

4 COMBINATIONS

Table S3 presents the outcomes achieved by each classifier when applied to the diverse combinations presented in Table 3. It should be noted that, for any given classifier, the optimal outcome was observed to be the combination of the response of the carp prompt both before and after the application of the unlearning algorithm.

Combination	Accuracy	Precision	Recall	F1 Score
**Naive Bayes (NB)**				
(3)+(4)	0.698	0.709	0.700	0.695
(5)+(6)	0.558	0.559	0.555	0.549
(3)+(5)	**0.719**	**0.770**	**0.725**	**0.709**
(4)+(6)	0.680	0.701	0.699	0.694
**Logistic Regression (LR)**				
(3)+(4)	0.674	0.675	0.673	0.673
(5)+(6)	0.698	0.708	0.695	0.692
(3)+(5)	**0.797**	**0.811**	**0.800**	**0.764**
(4)+(6)	0.674	0.674	0.674	0.674
**Support Vector Machine (SVC)**				
(3)+(4)	0.674	0.674	0.674	0.674
(5)+(6)	0.674	0.680	0.672	0.670
(3)+(5)	**0.797**	**0.811**	**0.800**	**0.796**
(4)+(6)	0.744	0.749	0.746	0.744

Table S3. Complete results of the combinations referenced in Table 3. Bold values indicate the best performance within each classifier.

REFERENCES

National Commission for the Protection of Human Subjects of Biomedical and Behavioral Research (1979). *The Belmont Report: Ethical Principles and Guidelines for the Protection of Human Subjects of Research*. Tech. rep., U.S. Department of Health, Education, and Welfare, Washington, D.C.

Zhong, Y. (2021). *WIKIBIAS: Detecting multi-span subjective biases in language*. Ph.D. thesis, The Ohio State University
